# Rheumatologists lack confidence in their knowledge of cannabinoids pertaining to the management of rheumatic complaints

**DOI:** 10.1186/1471-2474-15-258

**Published:** 2014-07-30

**Authors:** Mary-Ann Fitzcharles, Peter A Ste-Marie, Daniel J Clauw, Shahin Jamal, Jacob Karsh, Sharon LeClercq, Jason J McDougall, Yoram Shir, Kam Shojania, Zach Walsh

**Affiliations:** 1 Division of Rheumatology, Montreal General Hospital, McGill University Health Centre, 1650 Cedar ave, Montreal, Quebec H3G 1A4, Canada; 2 Alan Edwards Pain Management Unit, McGill University Health Center, Montreal, Canada; 3 Department of Anaesthesiology, Chronic Pain and Fatigue Research Center, University of Michigan Medical Center, Michigan, USA; 4 Division of Rheumatology, University of British Columbia, Vancouver, Canada; 5 Division of Rheumatology, The Ottawa Hospital, Ottawa, Canada; 6 Department of Medicine, University of Calgary, Calgary, Canada; 7 Departments of Pharmacology and Anaesthesia, Dalhousie University, Halifax, Canada; 8 Arthritis Research Centre of Canada, Vancouver, Canada; 9 Department of Psychology, University of British Columbia, Vancouver, Canada

**Keywords:** Cannabinoids, Rheumatic diseases, Physician knowledge

## Abstract

**Background:**

Arthritis pain is reported as one of the most common reasons for persons using medical herbal cannabis in North America. “Severe arthritis” is the condition justifying legal use of cannabis in over half of all authorizations in Canada, where cannabis remains a controlled substance. As champions for the care of persons with arthritis, rheumatologists must be knowledgeable of treatment modalities both traditional and non-traditional, used by their patients. As study of cannabinoid molecules in medicine is recent, we have examined the confidence in the knowledge of cannabinoids expressed by Canadian rheumatologists.

**Methods:**

The confidence of rheumatologists in their knowledge of cannabinoid molecules and mechanisms relevant to rheumatology, and their ability to advise patients about cannabinoid treatments was recorded by an online questionnaire circulated via email to the entire Canadian Rheumatology Association membership.

**Results:**

Over three quarters of the 128 respondents lacked confidence in their knowledge of cannabinoid molecules. While 45% of respondents believed there was no current role for cannabinoids in rheumatology patient care, only 25% supported any use of herbal cannabis. With 70% never having previously prescribed or recommended any cannabinoid treatment, uncertainty regarding good prescribing practices was prevalent. Concerns about risks of cannabis use were in line with the current literature.

**Conclusions:**

Rheumatologists lacked confidence in their knowledge of cannabinoid molecules in general and in their competence to prescribe any cannabinoid for rheumatic complaints. In line with this uncertainty, there is reticence to prescribe cannabinoid preparations for rheumatology patients. Guidance is required to inform rheumatologists on the evidence regarding cannabinoids.

## Background

Patients with rheumatic conditions almost universally experience pain with impact on quality of life. Chronic rheumatic pain is complex to treat in view of a dynamic interaction of various molecules and nerve pathways, all subject to nervous system plasticity. Drugs to treat pain offer mostly a modest effect, with use limited by adverse effects. In the absence of a cure for rheumatic pain, patients will continue to seek remedies to reduce symptoms.

Phytocannabinoids obtained from the plant *Cannabis sativa* have given symptom relief over centuries. With at least 66 different cannabinoid molecules in the plant *Cannabis sativa*, delta-9-tetrahydrocannabinol and cannabidiol are the best studied [1]. Musculoskeletal pain is commonly cited as a medical reason for using herbal cannabis, a dried preparation of *cannabis sativa*, in North America [2]. As the champions for rheumatic patient care, rheumatologists should be knowledgeable of current evidence for use of the category of cannabinoids, either pharmacological preparations termed synthetocannabinoids or the plant-derived compounds, phytocannabinoids, in rheumatic conditions to effectively advise patients.

With increasing patient advocacy to access herbal cannabis in the new millennium, the debate about decriminalization and possible legalization of cannabis has straddled the line between recreational and therapeutic use, with resulting regulatory changes in Canada and several states in the US. In Canada, cannabis has been legally available for medical purposes since 2001, following creation of an access program regulated by Health Canada. As of June 2013, almost 2/3 of the 32,000 Canadians who hold an authorization to possess medical cannabis had “severe arthritis” as the medical condition justifying use [3].

Recent changes in Canadian regulations will require health care professionals to prescribe herbal cannabis similarly to other prescriptions drugs. This accrued role for health care practitioners, likely to be followed in other countries, is controversial and has stimulated debate in the medical community [4,5]. To formally examine rheumatologists’ confidence about cannabinoids in general, and pertinence for use in rheumatic conditions, Canadian rheumatologists were surveyed.

## Methods

In March 2013, the entire Canadian Rheumatology Association (CRA) membership, approximately 510 rheumatologists, was invited via email to participate in a survey examining current knowledge and perceptions regarding cannabinoid use in rheumatology patients. The survey was available online from March 26th 2013 to April 5th 2013. A second reminder by email was sent to encourage non respondents. Data were collected once the survey was closed. The Canadian Rheumatology Association did not require ethical approval to carry out surveys of its members and ethical approval is not required for this type of study under Canadian law.

The 19-question survey comprised 3 sections. The first obtained demographic information, years and location of practice, type of practice (academic, community or private), and age and gender. The second addressed respondents’ current perception of knowledge of cannabinoids including phyto-, syntheto-, and endocannabinoids, as well as respondents’ belief whether there is a role for cannabinoids in rheumatology practice. The third addressed physicians’ response regarding interactions with patients requesting access to herbal cannabis or those in whom other pain treatments had failed.

Perception of knowledge of cannabinoids was assessed according to confidence (very confident; confident; somewhat confident; not confident). Respondents reported whether they had previously recommended trials of cannabinoid preparations to treat rheumatic pain, and whether they would consider a cannabinoid trial in the future.

Questions specifically pertaining to herbal cannabis were as follows: Do you believe there is a role for the use of medical cannabis in treating rheumatic diseases; how would you react if a patient asks about the use of medical cannabis; have you ever recommended a trial of medical cannabis or would you recommend a trial in the future; in the event of recommending medical cannabis, what is your confidence in writing a prescription regarding dosage, frequency of use and method of administration, and if a prescription was written what would be the starting dose, maximum dose, dose frequency and method of administration. Narrative responses were obtained for the following questions: variables needing consideration in patients requesting medical cannabis; advice given regarding precautions when using medical cannabis; patient characteristics that would be a barrier to a prescription of medical cannabis.

## Results

### Physician respondent demographics

The survey was sent to all 510 members of the CRA, with 128 (25%) responding. Demographic information for the respondents is shown in Table 1. The majority of respondents were over the age of 35, 57% were male, most had been in practice for over 10 years and were almost entirely practicing in urban areas. Over two thirds reported that their practice was academic or a combination of academic and private practice.

**Table 1:** Demographic information for 128 respondents, stratified according to confidence re: knowledge of cannabinoid molecules

**Characteristics**	**All**	**Not confident**	**Confident**
	**n** = **128**	**n** = **95**	**N** = **33**
Gender, out of 127 n (%)			
Male	72 (57)	48 (51)	24 (73)
Female	55 (43)	46 (49)	9 (27)
Age, out of 127 n (%)			
35 or younger	16 (13)	16 (17)	0 (0)
36–49	52 (41)	38 (40)	14 (42)
50 and over	59 (46)	40 (43)	19 (58)
Years in practice, out of 127 n (%)			
< 10	35 (28)	30 (32)	5 (15)
10–24	57 (45)	42 (45)	15 (46)
25 or over	35 (28)	22 (23)	13 (39)
Practice location n (%)			
Urban	119 (93)	87 (92)	32 (97)
Rural	9 (7)	8 (8)	1 (3)
Practice type n (%)			
Academic	46 (36)	33 (35)	13 (39)
Academic/private	37 (29)	28 (30)	9 (27)
Community hospital/private	24 (19)	19 (20)	5 (15)
Private	21 (16)	15 (16)	6 (18)

### Physician knowledge of cannabinoids and therapeutic considerations

Three quarters of respondents were not confident about their current knowledge of cannabinoid molecules (phyto, syntheto, and endocannabinoids), with only 18% reporting being somewhat confident. Lack of confidence in knowledge of the physiology of the endocannabinoid system in health and disease was reported by two thirds of respondents (see Table 2).

**Table 2:** Questions assessing confidence in 128 respondents

**Survey question**	**Response**			
“Do you feel confident regarding your current knowledge of the endocannabinoid system in health and disease?” (out of 126)	Very confident (0)	Confident (11)	Somewhat confident (29)	Not confident (86)
“Do you feel confident regarding your current knowledge of the cannabinoid to, syntheto, and endocannabinoids)?”	Very confident (0)	Confident (9)	Somewhat confident (24)	Not confident (95)
“How confident are you to write a prescription for medical cannabis indicating dosage, frequency of use, and method of administration?” (out of 127)	Very confident (1)	Confident (1)	Somewhat confident (10)	Not confident (115)

Almost half of respondents (45%) believed that there is currently no therapeutic role for use of any cannabinoid in the management of rheumatic diseases, whereas 25% believed there was a role for both pharmacologic cannabinoids and medical (herbal) cannabis, 25% believed there was a role for pharmacologic cannabinoids only, and 5% for medical (herbal) cannabis only.

Seventy percent of respondents had never previously recommended any form of cannabinoid treatment for patients, with 17% and 13% respectively having previously recommended pharmacologic preparations only, or pharmacologic and/or herbal preparations. Similarly, 60% of respondents would not currently recommend a trial of any cannabinoid preparation, with 20% each agreeable to recommend a trial of pharmacologic cannabinoids only, or pharmacologic and/or medical (herbal) cannabis.

### Specific responses regarding medical (herbal) cannabis

Seventy per cent of respondents believe that there is currently no role for medical (herbal) cannabis in the management of rheumatic diseases, with only 16 (13%) having ever previously recommended a trial. Over 90% of respondents were not confident in writing a prescription for medical (herbal) cannabis when required to indicate dosing, frequency, and method of administration. Eleven (9%) respondents indicated a starting dose of medical (herbal) cannabis, ranging from 2 puffs twice a day, to 0.5 g-3 g/day, with 9 (7%) identifying a ceiling dose, with the highest ceiling dose recommended as 5 g/day. A single dosing regimen was most commonly suggested by 6 (5%), with the rest suggesting a twice or three times a day dosing, with equal numbers recommending oral or inhaled administration. Reservations were stated regarding inhalation, but respondents acknowledged that herbal cannabis is mostly smoked. About half of respondents did not distinguish between medical (herbal) cannabis and pharmacologic cannabinoids with regard to efficacy (56%), side effects (53%) and treatment indications (60%).

When respondents described factors that require consideration for patients requesting medical (herbal) cannabis, the following were the most common variables identified by 74 respondents: previous substance abuse by 50, failed other treatments by 25, specific diagnosis by 15, mental health status by 14, patient age by 11, psychosocial issues and comorbidities by 9 each. Other variables mentioned less frequently included disease severity, smoking history, drug interactions, previous recreational use, doctor/patient relationship, diversion potential, and patient gender and occupation.

Forty respondents listed precautions that would be communicated to patients when prescribing medical (herbal) cannabis as follows: work and driving/operating machinery by 11, overuse/abuse and diversion by 11, addiction risk by 6, concurrent medication/substance use by 4, risks associated with smoking and cognitive impairment by 3 each, same precautions as for opioids by 2, and withdrawal, social support, cancer risk, weight gain as well as lack of scientific knowledge by 1 each. Eleven of the 40 admitted not knowing any precautions to communicate to patients.

When asked to list barriers that would prevent them from prescribing medical (herbal) cannabis, 47 respondents listed the following elements: History/potential of drug abuse or addiction by 27, mental health issues by 9, medical health including lack of clear diagnosis or non-severity of pain complaint by 9, and poor patient/physician relationship including history of non-compliance by 8. Other barriers noted less frequently were alcohol abuse, not having tried conventional treatments, smoking, criminal behaviour, diversion potential, risks related to driving and operating heavy machinery, and previous recreational use, amongst others.

### Grouping of respondents according to confidence in knowledge of cannabinoid molecules (results shown in Table 3)

When respondents were divided into those who reported confidence (n = 33) vs. non-confidence (n = 95) in their knowledge of cannabinoid molecules, 48% vs. 23% respectively believed that there is a role for medical (herbal) cannabis in the management of rheumatic diseases. Of those reporting confidence, 33% had previously recommended a trial of pharmacologic cannabinoids only, 27% a trial of medical (herbal) cannabis, whereas 39% had never recommended one or the other. In non-confident respondents, rates were respectively 12%, 7%, and 81%. One third vs. two thirds of confident vs. non-confident respondents would not recommend either pharmacologic cannabinoids or medical (herbal) cannabis in the future.

**Table 3:** Stratification of 128 respondents according to confidence and non-confidence in knowledge regarding cannabinoid molecules

	**Not confident**	**Confident**	**P value**
	**n** = **95**	**N** = **33**	
Is there a role for use of any of the following options for rheumatic conditions? n (%)			
Pharma only	21 (22)	10 (30)	NS
Medical cannabis	22 (23)	16 (48)	.008
No role	50 (53)	7 (21)	.0006
Did not answer	2 (2)	0 (0)	
Have you previously recommended a trial of the following? n (%)			
Pharma only	11 (12)	11 (33)	.007
Medical cannabis	7 (7)	9 (27)	.006
Never	77 (81)	13 (39)	< .0001
Would you recommend a trial of the following? n (%)			
Pharma only	15 (16)	10 (30)	NS
Medical cannabis	13 (14)	12 (36)	.009
None	65 (68)	11 (33)	.0008
Did not answer	2 (2)	0 (0)	
Would you write a medical cannabis prescription for a patient failing conventional treatments? n (%)			
Yes	22 (23)	14 (42)	.043
No	70 (74)	17 (52)	.029
Did not answer	3 (3)	2 (6)	
Would you write a medical cannabis prescription based on patient request regardless of previous treatments? n (%)			
Yes	2 (2)	3 (9)	NS
No	90 (95)	28 (85)	NS
Did not answer	3 (3)	2 (6)	
Confidence level regarding writing a medical cannabis prescription (dose, frequency) n (%)			
Expressed confidence	3 (3)	9 (27)	.0002
Not confident	92 (97)	23 (70)	< .0001
Did not answer	0 (0)	1 (3)	

When conventional treatments have failed, 42% vs. 23% of confident vs. not confident respondents would write a prescription of medical (herbal) cannabis, whereas only 9% vs. 2% respectively would write a prescription for medical (herbal) cannabis on the basis of patient request. Even amongst confident respondents, less than one third indicated confidence in writing a prescription for medical (herbal) cannabis when required to specify dosage, frequency of use, and method of administration.

## Discussion

This study demonstrates lack of confidence by rheumatologists in their knowledge of cannabinoids in general and in their ability and competence to prescribe any cannabinoid, either pharmacological or herbal preparations for rheumatic pain. In line with this uncertainty, there is an overall reticence in providing prescriptions for any cannabinoid preparation for rheumatology patients. These observations should raise two important concerns. Firstly, as cannabinoids are known to have a physiologic role in the human organism, albeit not yet fully defined, this shortfall in basic knowledge of a system with pertinence in rheumatic conditions indicates a knowledge gap between current science and the clinician. Secondly, in the setting of increased public demand for access to herbal cannabis for chronic conditions, there exists a mismatch between patient needs and advocacy,and physician knowledge [6]. This lack of knowledge likely stems from the paucity of clinical information regarding use of cannabinoids in rheumatology practice [7]. This societal groundswell is however reflected by regulatory bodies worldwide that are considering the merits of legalizing herbal cannabis.

The current controversy about cannabinoid treatments centers primarily on the role of herbal cannabis (“medical marijuana”) used as a therapeutic modality, particularly in the context of rheumatic pain management. To date there is not a single controlled clinical study that has examined efficacy or safety of herbal cannabis in the rheumatic diseases [7]. Pharmacological cannabinoids such as nabilone are legal and readily available in many countries, and research on their safety and efficacy, while still modest, is considerably more advanced than research on herbal cannabis. Furthermore, pharmacological cannabinoids have a more reliable and safe delivery system, whereas administration of herbal cannabis, which is often smoked, presents an uncontrolled delivery system with potential harm due to inhaled toxic substances. There is however increasing evidence for harm when herbal cannabis is used, including acute psychomotor effects leading to accidents, and long term effects on cognition, respiratory function and risk of addiction.

The ubiquitous nature of the endocannabinoid system raises questions as to the exact function of this system in health and disease, with preclinical studies contributing to the understanding of physiologic mechanisms, but with limited experience in the clinical arena. Contrary to popular belief, the cannabinoid effects are not only confined to the nervous system and pain pathways, but have effects on inflammation and joint damage [8]. This highlights a promising role for cannabinoids in the management of various rheumatic conditions, but with need for sound basic science and clinical study.

The overriding principle for effective rheumatic pain management is symptom relief with maintained function, rather than palliation [9]. Although numerous non-pharmacologic and pharmacologic strategies are available, pain treatments remain suboptimal [10]. Treatment of musculoskeletal pain is a common reason for use of herbal cannabis. For example, 80% of users in a pain clinic in the US reported myofascial pain, and up to one third of persons in two population studies in the UK and Australia reported using cannabis for treatment of arthritis pain [2,11,12]. In the state of Colorado, with 2% of the population registered to use medical herbal cannabis, pain although not further specified, is the reason for use in over 90% of persons [13].

The absence of any recommendation for use of any cannabinoid, and particularly herbal cannabis for treatment of arthritis pain in current treatment guidelines likely contributes to the low rate of confidence expressed by participants of this survey. It is therefore understandable that rheumatologists report limited previous experience in prescribing any form of cannabinoid molecule, with less than a quarter willing to recommend herbal cannabis. Among confident respondents, 27% had recommended a trial of herbal cannabis, compared to only 7% of non-confident respondents. In the absence of clinical evidence to support a role for herbal cannabis in the management of rheumatic pain, rheumatologists’ reluctance to prescribe is entirely appropriate. It also follows that those who are most confident in their knowledge of cannabinoids are also more likely to prescribe herbal cannabis.

Even those who would suggest use of herbal cannabis and were confident in their knowledge of cannabinoids expressed lack of knowledge of dosing and methods of administration. The results of this study are in line with concerns expressed by family physicians in Colorado [13]. Similar to the opinions of Canadian rheumatologists, only 19% of family physicians who responded to a questionnaire thought that medical marihuana should be recommended to patients. This is of particular interest as Colorado is the US state with the greatest per-capita persons registered to possess herbal cannabis for medicinal use. Therefore both these surveys of North American physicians demonstrate an important gap between public and legislative perceptions and the comfort of the medical community in advising patients.

In Canada, the medical cannabis access program governed by Health Canada was repealed and replaced by the *Marihuana for Medical Purposes Regulations* in April 2014, with health care practitioners bearing full responsibility for providing a prescription for herbal cannabis. If physicians are to prescribe herbal cannabis, medical ethics and deontology require that they be competent regarding the prescribed treatment (molecules, dosage, safety, etc.), which is not the case as demonstrated by this survey. This is particularly concerning as “severe arthritis” is cited as the most common diagnosis for persons holding an authorization to possess cannabis in Canada [3].

There is therefore an evident disconnect between patients’ needs and good medical practice regarding the prescription of cannabinoid molecules in general. This disconnect likely explains the “awkward” strain put on the physician-patient relationship, particularly when the subject of herbal cannabis is broached, with concerns that new regulations may further exacerbate this strenuous conversation [5].

Although this study reflects the beliefs of rheumatologists from a single country, it is noteworthy that Canada currently has liberal regulations regarding medical herbal cannabis access. Our study is also limited by the response rate of 25%, however this rate is in keeping with the usual response rates of surveys in general, and reasons for non-response may be explained by apathy, lack of knowledge or disagreement with cannabinoid use for rheumatic conditions.

## Conclusions

The results of this survey, whereby the greater proportion of respondents expressed insecurity regarding cannabinoid knowledge, has highlighted the need for more in depth evaluation of both phyto- and synthetocannabinoids in rheumatic disease management, as well as education for the health care community. Further research should address factors such as the specific cannabinoid molecules that could be used, dosing, method of administration, side-effects, and drug interactions, as well as the specific rheumatic conditions for which cannabinoids could be prescribed. However, even in the absence of substantial scientific proof, the health care community will require guidance regarding the use of cannabinoids in the treatment of rheumatic conditions in view of growing public advocacy and patient requests.

## Competing interests

The authors declare that they have no competing interests.

## Authors’ contributions

MAF, PAS, SJ, JK, SL, JJM, YS, KS and ZW Participated in the questionnaire design, analysis of the data and writing of the manuscript. DJC participated in analysis of the data and writing of the manuscript. All authors read and approved the final manuscript.

## Pre-publication history

The pre-publication history for this paper can be accessed here:

http://www.biomedcentral.com/1471-2474/15/258/prepub
